# Introgression of bruchid (*Zabrotes subfasciatus)* resistance into small red common bean ( *Phaseolus vulgaris* ) background and validation of the BRU_00261 (snpPV0007) resistance marker

**DOI:** 10.1111/pbr.12969

**Published:** 2021-10-09

**Authors:** Shiferaw Girsil Tigist, Bodo Raatz, Amelework Assefa, Rob Melis, Julia Sibiya, Gemechu Keneni, Clare Mukankusi, Berhanu Fenta, Selamawit Ketema, Dagmawit Tsegaye

**Affiliations:** ^1^ Ethiopian Institute of Agricultural Research Melkassa Agricultural Research Centre Adama Ethiopia; ^2^ Bean Program International Centre for Tropical Agriculture (CIAT) Cali Colombia; ^3^ Vegetable and Ornamental Plant Agricultural Research Council Pretoria South Africa; ^4^ African Centre for Crop Improvement University of KwaZulu‐Natal Pietermaritzburg South Africa; ^5^ Holeta Agricultural Research Centre Ethiopian Institute of Agricultural Research Addis Ababa Ethiopia; ^6^ Beans Program International Centre for Tropical Agriculture (CIAT) Kampala Uganda

**Keywords:** arcelin gene, bruchids, common bean, Mexican bean weevil, molecular marker

## Abstract

Bruchids are a major storage pest of common bean. Genetic resistance is a suitable method to avoid grain losses during storage. The objective of the study was to introgress the arcelin‐based resistance locus into selected advanced breeding line and to validate the molecular marker BRU_00261. A total of 208 progeny F_4_ families were phenotyped using a randomized complete block design, with three replications. Highly significant differences (*P* <  .001) among the entries, parents and offspring were recorded for almost all traits. There was no significant difference between the two parents in the number of eggs laid. The progenies were grouped as highly resistant (34.3%), resistant (11.9%), moderately resistant (21.4%) and susceptible (32.4%). The levels of broad sense heritability ranged from 68.5%–93.9% for all the traits. Eighty‐three most resistant lines and the parental lines were genotyped with the marker BRU_00261 (snpPV0007). The marker segregation deviated significantly from the expected independent segregation towards a strong enrichment for the resistant marker in the selected families. This marker will be useful for selecting promising materials in early generations and phenotypic confirmation of positive lines in later generations.

## 1 INTRODUCTION

The common bean is one of the most important legumes worldwide and serves as part of the daily diet for more than 300 million people (Lioi & Piergiovanni, 2013). In eastern and southern Africa, it is the second most important dietary protein source (Cardona & Kornegay, 1999). Despite its importance, the crop is affected by various biotic and abiotic stress factors. Of the biotic factors, the storage insect pests *Zabrotes subfasciatus* (Boheman) and *Acanthoscelides obtectus* (Say) are the most destructive post‐harvest pests (Cardona, 2004; Cardona et al., 1992). The grain damage caused by the insects reduces the quality, marketability, germination and seedling vigour (Blair, Muñoz, et al., 2010). In developing countries, the two bean bruchid species cause up to a 13% loss in stored bean grain (Cardona & Kornegay, 1999). In the warmer regions of East and Central Africa, *Z. subfasciatus* is the most common (Nchimbi & Misangu, 2002; Tadesse et al., 2008) storage pest. In the Central Rift Valley of Ethiopia, where the common bean is one of the major crops, *Z. subfasciatus* is considered to be a serious storage pest that causes immense damage on stored beans (Getu et al., 2003; Negasi, 1994; Wortman et al., 1998).

The Mexican bean weevil (*Z. subfasciatus*) is more prevalent in the lower altitudes (<1,000 m above sea level) and warmer areas. Hence, *Z. subfasciatus* is more important in the tropical and subtropical regions (Hill, 1983). The infestation and damage by *Z. subfasciatus* occur only in storage (Schoonhoven, 1976), and most of the cultivars and landraces are susceptible to the pest (Schoonhoven & Cardona, 1982). Very few wild common bean genotypes, which originate from the central highlands of Mexico, were found to be resistant (Acosta‐Gallegos et al., 1998; Schoonhoven et al., 1983). One mechanism of resistance is believed to be antibiosis, which is conferred by the seed storage proteins produced by the APA (arcelin, phytohemagglutinin and α‐amylase inhibitor) gene family (Acosta‐Gallegos et al., 1998; Schoonhoven et al., 1983). The antibiosis exhibited through insecticidal activities affects the emergence of adult bruchids and hampers insect growth and development (Osborn et al., 1988).

Although the APA proteins differ in their biochemical and physiological properties, their expression shows similar patterns (Moreno et al., 1990). Based on protein size and electrophoresis patterns, arcelin is different from all the other APA proteins (Romero‐Andreas et al., 1986). Several variants of arcelin were identified, each with a different effect on *Z. subfasciatus*. Currently, eight variants of arcelin (Arc‐1 to Arc‐8), with different levels of resistance have been identified from wild accessions of the common bean (Acosta‐Gallegos et al., 1998; Lioi & Bollini, 1989; Osborn et al., 1986; Santino et al., 1991; Zaugg et al., 2012). Arc‐5 and Arc‐1 showed the highest level of resistance to *Z. subfasciatus*, followed by Arc‐4, Arc‐2, and Arc‐3, in their order of importance (Cardona et al., 1990). The resistance to Zabrotes (RAZ) lines developed by CIAT, using Arc‐1 variant lines through backcrossing (Cardona et al., 1990), and marker‐assisted Zabrotes (MAZ) lines developed by crossing RAZ lines with different genotypes were screened for resistance in a previous study. Several RAZ and MAZ resistant lines were identified, although all the commercial varieties, landraces and advanced breeding lines from CIAT were susceptible to the insect (Tigist et al., 2018).

Traditionally, selection for resistant lines is done through laboratory screening, which has been found to be tedious and time‐consuming and requires large laboratory space and a large amount of seed, to undertake replicated trials. Marker‐assisted selection (MAS), on the other hand, has been shown to be a simpler and more efficient (cost effective and technically simpler) tool in the development of bruchid‐resistant cultivars (Miklas et al., 2006). DNA‐based markers have been applied, to monitor the expression of the arcelin protein in breeding programmes (Miklas et al., 2006). Several attempts have been made to identify molecular markers that are tightly linked to the arcelin gene (Miklas et al., 2006; Blair, Muñoz, et al., 2010). The arcelin genes were mapped on chromosome 4 of the common bean genome (Nodari et al., 1993). Based on populations developed from crosses between the RAZ lines and susceptible varieties, several simple sequence repeat (SSR) markers associated with the arcelin gene were identified (Blair et al., 2002; Blair, Muñoz, et al., 2010). SNP markers that are linked to the arcelin locus were later designed on chromosome 4 based on positional information from Blair, Muñoz, et al. (2010) (Raatz et al., 2019). The newly developed SNP markers should be useful for MAS and the introgression of arcelin, to develop bruchid‐resistant lines for effectiveness and efficiency of the breeding programme. Therefore, the objective of the study was to introgress arcelin‐based resistance locus into selected advanced breeding line and to validate the newly developed marker BRU_00261 (snpPV0007) in the SCR15 × MAZ200 population.

## 2 MATERIALS AND METHODS

### 2.1 Plant material and crosses

Based on the level of resistance to bruchid (*Zabrotes subfasciatus*) infestation, agronomic suitability and participatory variety selection, two parental materials were selected from the small red bean (*Phaseolus vulgaris* L.) grain market class. The lines included ‘SCR15’, a small red variety released in 2017, and MAZ200, a bruchid‐resistant line identified by Tigist et al. (2018). MAZ200 is derived from a cross between Mesoamerican bruchid resistance source RAZ168 and breeding line RMA68. RAZ lines were the result of a pre‐breeding effort to produce bruchid‐resistant lines with acceptable grain types and agronomic performance. MAZ lines were generated with the intention to obtain more elite lines with bruchid resistance. SCR15 is a drought tolerant line but is highly susceptible to bruchid attack (Tigist et al., 2018). The crosses were conducted at Melkassa Agricultural Research Center in the screen house, to avoid natural insect and disease infestation. The F_1_ plants were selfed, in order to generate enough seed for the subsequent experiment.

### 2.2 Laboratory phenotyping

Two hundred eight F_2:3_ families derived from single F_2_ plants were planted for advancement, and F_2:4_ seeds were harvested in bulk to get enough seed for laboratory phenotyping. The 208 families were phenotyped in the Entomological Research Laboratory at the Melkassa Agricultural Research Center, Ethiopia, under ambient temperature and humidity. To supply bruchids of a similar age for the experiment, a culture of bean bruchid was developed. A susceptible variety was used for the culture, and adult bruchids collected from the stored bean seed were used to develop the culture. The mass rearing of bruchid was carried out under an average room temperature of 27°C and a relative humidity of 70%.

Based on the protocol by Dobie (1977), freshly harvested seeds were cleaned and placed in a deep freezer (−20°C) for 4 weeks to destroy any prior bruchid infestation. The seeds were kept under room temperature in the laboratory for 7 days. Thereafter, 15 seeds were placed in a transparent plastic jar of 6 cm × 7 cm size. To provide for proper ventilation, the lids of the plastic jars were perforated and covered with mesh that had a small pore size, to prevent the bruchids from escaping. Each jar was infested with three pairs (female and male) of newly emerged bruchids. The jars that contained the bean seeds and bruchids were laid out in the laboratory, in a randomized complete block design (RCBD), with three replications. The jars were incubated for oviposition. After 10 days, the parental bruchids were removed, and the number of eggs laid was counted. The jars were monitored on a daily basis, to observe for any progeny bruchid emergence. From the first appearance of bruchid progeny, the jars were monitored every 2 days, for recording purposes, and for the removal of newly emerged bruchids.

Data were recorded on the number of eggs per adult female (NE), the number of adult bruchids emerged (NAE) and seed damaged (% of seed with holes) (PD). The percentage adult weevil emergence (PAE) was calculated, based on the total number of adults emerged compared with the number of eggs laid. Percentage grain weight loss was estimated using count and weight method (Gwinner et al., 1990):

$$\text{Weight loss}\;\left(\%\right)=\frac{\left(\mathrm{Wu}*\mathrm{Nd}\right)-\left(\mathrm{Wd}*\mathrm{Nu}\right)}{\mathrm{Wu}\left(\mathrm{Nd}+\mathrm{Nu}\right)}*100,$$

where Wu is the weight of undamaged grain, Wd is the weight of damaged grain, Nu is the number of undamaged grain, and Nd is the number of damaged grains.

The genotypes were classified, based on the percentage adult emergence as described by Blair, Prieto, et al. (2010). The genotypes with adult emergence from 0% to 15% were classified as highly resistant (HR), those from 15% to 30% as resistant (R), those from 30% to 50% as intermediate resistance (IR) and those from 50% to 100% as susceptible (S).

To ensure the homogeneity of variance, the data, based on count and percentage values were transformed, using natural log and arcsine transformation, respectively. The phenotypic data were subjected to statistical analysis, using GenStat Version 18 (Payne et al., 2017). The variance components were estimated, using the REML tool in GenStat Version 18. During the analysis, both the genotypes and replications were considered as random effects. Boxplot and phenotypic correlation were generated using ggplot2 and ggcorrplot package in R version 3.6.2.

Heritability, in a broad sense, was estimated as follows:

$$H^{2}=\frac{\sigma^{2}G}{\sigma^{2}P.},$$

where *σ* ^2^ *G* is the genotypic variance and *σ* ^2^ *P* is the total phenotypic variance.

The coefficient of variation was calculated as follows:

$$\mathrm{CV}=\frac{\sigma }{\mu }\times 100,$$

where *σ* is the standard deviation and *μ* is the mean.

The phenotypic segregation was analysed to evaluate if the data fits the segregation ratio for a single gene locus using the formula:

$$X^{2}=\frac{\sum {\left(O-E\right)}^{2}}{E},$$

where *O* is the observed frequencies and *E* is the expected frequencies.

### 2.3 Validation of the BRU marker in the SCR15 × MAZ200 population

Eighty‐three most resistant F_2:4_ family (72 highly resistant group and 11 from the resistant group with PAE 15%–20%) seed were selected and planted at Melkassa Agricultural Research Center. Leaf discs from young leaves of these lines and their resistant and susceptible parents were collected from a single‐bean plant per line. Leaf discs were collected, dried and shipped to the genotyping provider for DNA extraction and SNP assay genotyping to Intertek, Sweden. These lines and the parental lines were genotyped using the genotyping outsourcing providers Intertek (www.intertek.com), who evaluated 10 KASP markers for each line (Table S1). Among the 10 markers, the marker of interest was BRU_00261 (Intertek ID: snpPV0007) that is located in the arcelin locus on the end of chromosome 4 (Mukankusi et al., 2019). This marker previously described by Raatz et al. (2019) was designed using positional information on the arcelin locus from Blair, Muñoz, et al. (2010). Sequencing data from a MAZ line in the vicinity of previously identified arcelin markers were used to identify SNPs that were subsequently confirmed in several MAZ and RAZ lines.

## 3 RESULTS

### 3.1 ANOVA and heritability estimates

The mean squares for five bruchid infestation response traits are shown in Table 1. The analysis of variance (ANOVA) revealed highly significant differences (*P* <  .001) among the genotypes for all the traits. Significant differences between the two parents were high for the traits number of adults emerged and the percentage adult emergence (*P* <  .001) and also significant for the percentage seed damage and the percentage seed weight loss (*P* < .01), whereas there was no significant difference between parents for the number of eggs (*P* >  .05).

**TABLE 1:** Analysis of variance (ANOVA) for five bruchid infestation response characters studied on 208 F_4_ families and parental genotypes

Source of variation	DF	Mean squares				
		NE	NAE	PAE	PD	SWL (%)
Rep	2	139.80	157.38	526.70	552.20	11.10
Lines	209	622.10**	402.41**	2342.60**	2335.10**	174.71**
Parents	1	860.70^ns^	2742.00**	7351.30**	2722.90*	310.11*
Offspring	207	624.80**	384.09**	2288.40**	2320.40**	172.62**
Residual	418	195.90	36.63	130.60	140.20	18.98

Abbreviations: DF, degrees of freedom; NAE, number of adult emerged; NE, number of eggs; ns, non‐significant; PAE, percentage adult emergence; PD, percent seed damage; SWL (%), seed weight loss.

* Significant at *P* = .01.

** Significant at *P* < .001.

The mean, minimum, maximum, heritability and coefficient of variation values for five bruchid infestation response parameters are presented in Table 2. The number of eggs laid ranged from 3.0 to 114.0, with a mean of 35.7 eggs per genotype. However, the number of adults emerged ranged from 0.0 for the resistance genotypes, to 58.0 for the highly susceptible genotypes, with a mean of 12.0 adults per genotype. Similarly, out of the total eggs laid on each genotype, none of the eggs (0.0%) hatched on the resistant genotypes, whereas all the eggs (100.0%) hatched into adults on the highly susceptible genotypes, with a mean percentage adult emergence of 34.3 per genotype. Percentage seed damage and seed weight loss ranged from 0.0% to 93.3% and 0.0% to 33.8%, respectively. The coefficient of variation calculated for the five parameters among the 210 genotypes ranged from 12.2% for number of eggs to 42.7% for percentage seed damage. Broad sense heritability recorded was high in all cases indicating good reproducible data quality. The lowest value was found for the number of eggs (68.54%), whereas all others were found in a high range from 89% to 94%.

**TABLE 2:** Summary statistics on five response characters evaluated on 208 F_4_ families' and parental genotypes under bean bruchid infestation

Trait	Min	Max	Mean	H^2^ (%)	CV (%)
Number of eggs	3.00	114.00	35.67	68.54	12.2
Number of adult emerged	0.00	58.00	12.02	89.96	20.2
Percentage adult emergence	0.00	100.00	34.29	93.93	26.2
Percentage seed damage	0.00	93.30	32.75	93.68	27.5
Percentage seed weight loss	0.00	33.80	11.33	88.72	27.5

Abbreviations: CV, coefficient of variation; H^2^, broad sense heritability.

### 3.2 Phenotypic performance of parents and offspring

**FIGURE 1:**
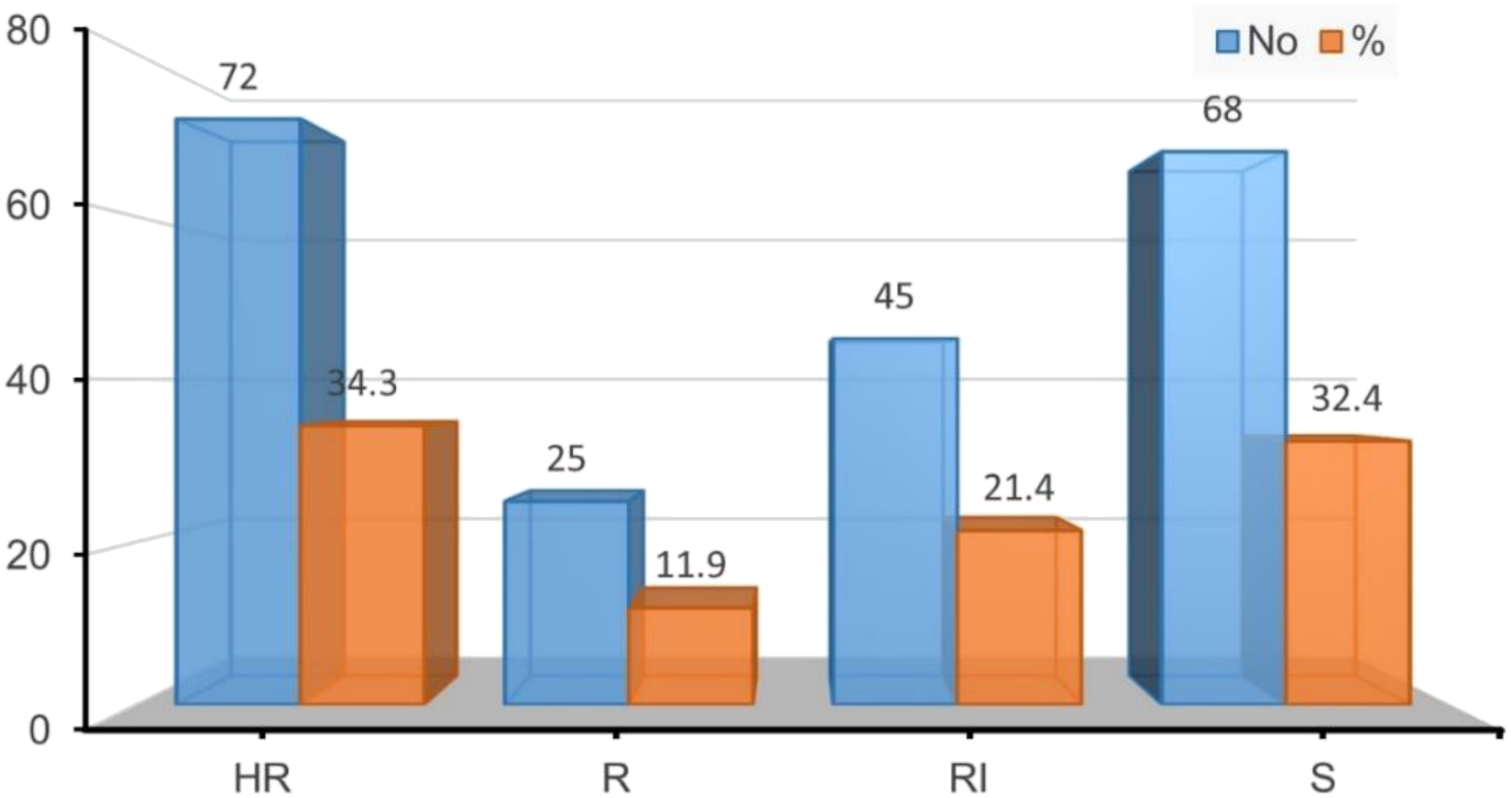
Classification of progeny genotypes, based on bruchid susceptibility classes using percentage adult emergence: HR, highly resistant; R, resistant; RI, intermediate resistant; S, susceptible F_4_ families [Color figure can be viewed at wileyonlinelibrary.com]

On the basis of percentage adult emergence, the progenies were classified into four classes. Genotypes were categorized as highly resistant to susceptible. Out of the 208 lines and two parents, 34.3% of the progenies were found to be highly resistant, 11.9% resistant, 21.4% moderately resistant and 32.4% susceptible (Figure 1).

**FIGURE 2:**
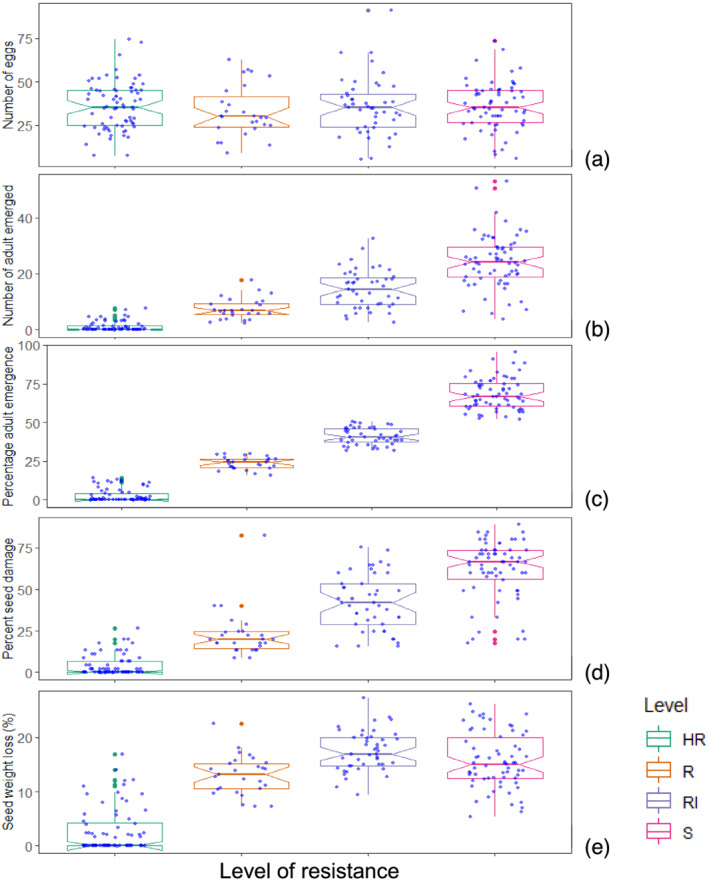
Boxplots of five bruchid response traits evaluated in 208 lines grouped by level of resistance. Level of resistance abbreviations are as follows: HR, highly resistance; R, resistant; RI, intermediate resistant; S, susceptible [Color figure can be viewed at wileyonlinelibrary.com]

A box plot of five traits by resistance level revealed that there was no variability for number of eggs between resistance levels (Figure 2). Although a high number of eggs were laid in the highly resistant genotype category, less than 15% of the eggs emerged into adults and the percentage seed damage and percentage seed weight loss ranged from 0.0% to 26.7% and 0.0% to 16.9%, respectively. In the susceptible genotypes, about 95% of the laid eggs hatched into adults and caused significant seed weight loss. As the number of emerged adults increased, the percentage of seed damage and seed weight loss increased. In the present study, the resistant parent MAZ200 was grouped in the highly resistant category and 40 of its offspring revealed a similar resistant response, with a zero percentage adult emergence (Table 3). The highly susceptible parent ‘SCR15’ revealed a maximum percentage adult emerged (Table 4), and 50 offspring showed high percentage adult emergence (>60%). The top 10 best performing and susceptible lines are presented in Tables 3 and 4, respectively. A *χ* ^2^ value of 32.16 indicated a significant deviation from the expected independent segregation in F_2:4_ and the observed ration, assuming Classes 2 and 3 to correspond to the heterozygous state (Figure 1).

**TABLE 3:** Mean responses to bruchid infestation among the top 10 highly resistant F_4_ families

Offspring lines	NE	NAE	PAE	PD	PSWL
SCR15/MAZ200‐115	74.7	0.0	0.0	0.0	0.0
SCR15/MAZ200‐104	72.7	0.0	0.0	0.0	0.0
SCR15/MAZ200‐191	65.7	0.0	0.0	0.0	0.0
SCR15/MAZ200‐131	53.7	0.0	0.0	0.0	0.0
SCR15/MAZ200‐129	52.3	0.0	0.0	0.0	0.0
SCR15/MAZ200‐135	51.7	0.0	0.0	0.0	0.0
SCR15/MAZ200‐168	51.7	0.0	0.0	0.0	0.0
SCR15/MAZ200‐188	48.7	0.0	0.0	0.0	0.0
SCR15/MAZ200‐107	46.3	0.0	0.0	0.0	0.0
SCR15/MAZ200‐121	46.0	0.0	0.0	0.0	0.0
MAZ200 (resistant parent)	48.7	0.0	0.0	0.0	0.0
SCR15 (susceptible parent)	55.7	53.0	95.3	60.0	20.2

Abbreviations: NAE, number of adult emerged; NE, number of eggs; PAE, percentage adult emergence; PD, percent seed damage; SWL (%), seed weight loss.

**TABLE 4:** Mean responses for bean bruchid infestation characters among the 10 highly susceptible F_4_ families

Offspring lines	NE	NAE	PAE	PD	PSWL
SCR15/MAZ200‐080	32.7	29.7	91.0	8.0	15.5
SCR15/MAZ200‐051	34.3	30.3	88.3	68.9	10.1
SCR15/MAZ200‐113	32.7	28.7	88.3	73.3	13.6
SCR15/MAZ200‐109	24.7	20.7	83.8	77.8	18.0
SCR15/MAZ200‐175	37.7	29.3	83.6	71.1	20.4
SCR15/MAZ200‐097	62.0	50.7	82.4	84.4	9.8
SCR15/MAZ200‐119	21.7	17.3	80.0	48.9	24.7
SCR15/MAZ200‐071	24.7	19.7	79.4	68.9	11.6
SCR15/MAZ200‐154	20.7	16.3	79.3	42.2	23.3
SCR15/MAZ200‐065	42.7	33.7	78.6	84.4	5.4
SCR15 (susceptible parent)	55.7	53.0	95.3	60.0	20.2
MAZ200 (resistant parent)	48.7	0.0	0.0	0.0	0.0

Abbreviations: NAE, number of adult emerged; NE, number of eggs; PAE, percentage adult emergence; PD, percent seed damage; SWL (%), seed weight loss.

### 3.3 Correlation between traits

**FIGURE 3:**
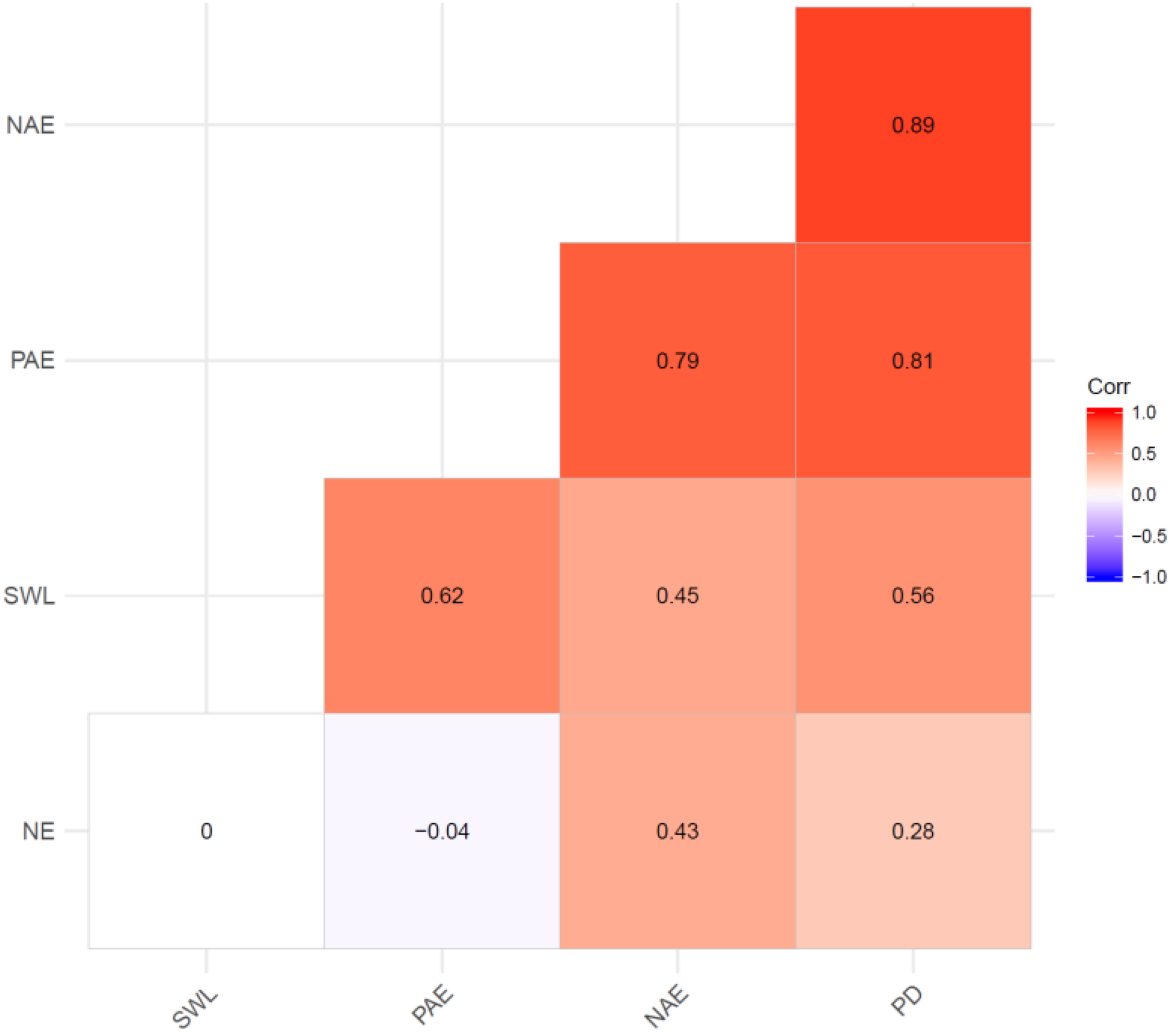
Phenotypic correlations among different response characters to bean bruchids infestation in 85 common bean lines: NE, number of egg; NAE, number of adults emerged; PAE, percentage adult emergence; PD, percent seed damage; SWL, seed weight loss [Color figure can be viewed at wileyonlinelibrary.com]

The relationships between the five different traits showed mostly positive strong associations (Figure 3). Number of eggs showed no significant correlation with seed weight loss and percentage adult emergence but positive and significant relationship with number of adults emerged. Seed weight loss had a strong relationship with other traits except number of eggs. The trait percentage adult emergence also had a very strong relationship with number of adults emerged and percent seed damage. The trend of relationship between number of adults emerged and percent seed damage was similarly strong and positive.

### 3.4 Molecular marker validation

**FIGURE 4:**
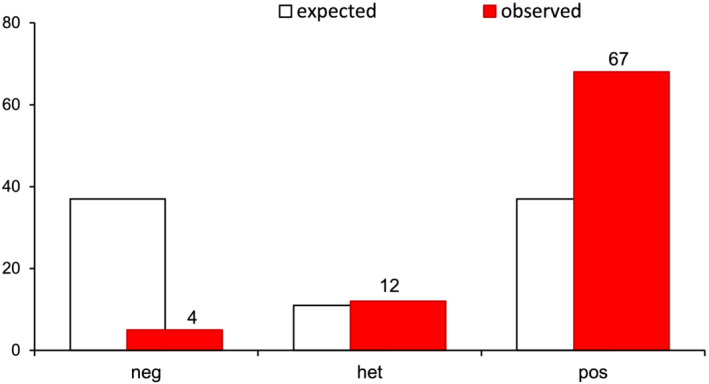
Expected and observed segregation for the bruchid resistance marker BRU_00261 tagging the arcelin locus in the 83 F_4_ lines of the SCR15 × MAZ200 population selected for bruchid resistance [Color figure can be viewed at wileyonlinelibrary.com]

Eighty‐three most resistant lines and the parental lines were sown and genotyped with the marker BRU_00261 (snpPV0007) using a genotyping outsourcing service (Table S1 and Figure S1). In the selected families, the marker segregation deviated significantly from the ratio expected for independent segregation in F_4_ (7:2:7, or in a population of 83 lines: 36 homozygous resistant, 11 heterozygous, and 36 homozygous susceptible). Instead, a strong enrichment for the resistant marker allele was observed (67:12:4) (Figure 4). Most resistant lines were homozygous for the marker, indicating a strong correlation between the genotype at this locus and bruchid resistance. As the most resistant 40% (83 out of 208) of F_2_‐derived families were selected, a few heterozygous and susceptible genotype calls were also observed in these F_4_ individuals. The observation that phenotypic selection led to an enrichment of the marker means that the marker can be employed in selection in this germplasm to increase resistance.

## 4 DISCUSSION

The highly significant mean squares for all the bruchid response traits recorded in this study suggest the presence of a strong genetic variation. The parents also revealed significant differences (*P* <  .01) for most traits, indicating that the parents were very diverse in relation to their level of resistance to bean bruchid. The non‐significant difference in the number of eggs between the two parents confirmed that the mechanism of resistance of the arcelin gene is antibiosis, which is characterized by delaying adult emergence and hampering insect growth and development (Osborn et al., 1988). The lack of correlation between the number of eggs laid and the number of adults emerged was also reported by other similar studies (Negasi & Abate, 1992; Shiferaw, 2004). In the present study, it was recorded that, out of the 56 eggs laid, on average, 53 hatched into adults (95%) in the susceptible parent (‘SCR15’). However, in the resistant parent (MAZ200), out of the 49 eggs laid, none of the eggs hatched into adults. This indicates that the parents differentiated well in their response to bruchid infestation. Similar results were also reported by Blair, Prieto, et al. (2010) for the arcelin gene.

For an efficient and effective breeding programme, it is important to know the genetic variability, by using appropriate parameters, such as heritability estimates (Atta et al., 2008). In the present study, generally high levels of heritability values were recorded, which means that a greater proportion of the phenotypic variation observed in this study was explained by the genotypic variations among genotypes. Because the large heritability were noted for most parameters except number of eggs, selection based on these would be effective towards improving bean bruchid resistance. The high heritability values reported by Kasozi (2013) for the susceptibility parameters, such as weevil progeny emergence and percent grain damage on the maize weevil (*Sitophilus zeamais*) were consistent with the present results. However, Keneni (2012) in his study on adzuki bean beetle (*Callosobruchus chinensis* L.) on chickpea reported low heritability values (0.20%–11%) for all insect‐related traits, such as the number of eggs, the number of adult emerged and the percentage adult emergence and high heritability (76%) for seed weight loss in the same experiment.

The correlation between most of the susceptibility traits (number of adults emerged, percentage of adults emerged, percent seed damage and seed weight loss) were positive and significant. Therefore, the seed weight loss caused by bean bruchid can be reduced by selection of genotypes with reduced adults emerged and percentage of adults emerged and genotypes with low percent damage and seed weight loss. The same pattern of associations with some exceptions was reported on common bean and cowpea infested by *C. maculatus* (Mwila, 2013; Redden & McGuire, 1983), on chickpea infested by *C. chinensis* (Aslam et al., 2006; Keneni, 2012) and on maize infested by *Sitotroga cerealella* and *S. zeamais* (Tefera et al., 2013; Demissie et al., 2015).

The BRU_00261 (snpPV0007) marker showed a strong enrichment for the arcelin locus after phenotypic selection for bruchid resistance, hence validating its utilization in MAB programs. Only a few selected lines (~5%) did not show the resistant alleles. Non‐resistant genotypes at a low frequency are expected as individuals of segregating F_2_derived F_4_ families were among those genotyped and also because phenotypic accuracy has its limits. This result suggests this marker to be a valuable tool for selecting promising materials in early generations, although a phenotypic confirmation of positive lines in later generations is suggested.

## 5 CONCLUSIONS

The introgression of the arcelin locus into a small red bean background line was effective in the case of SCR15 × MAZ200 population. Within the 208 segregating progeny population, half of the progenies were found to be resistant or highly resistant using phenotypic screening. Natural infestation and damage of *Z. subfasciatus* only occurs in storage starting from pre‐existing bruchid populations. All genotypes were equally exposed to the same age, and number of insects and the laboratory conditions were conducive to insect growth and development. This type of screening is standard procedure for storage pests, and the outcome is conclusive, because the test is a non‐choice test. In addition, the parameters used to screen the population revealed high levels of heritability, indicating that the considerable variations among the progeny population may be due to the genes. This, in turn, suggests that selection should be effective for developing new varieties with high levels of bruchid resistance. The use of MAS in early generation will increase the efficiency of breeding for bruchid resistance.

## CONFLICT OF INTEREST

The authors have no any conflict of interest to declare.

## AUTHOR CONTRIBUTIONS

Tigist Girsil involved in designing and performing the experiments, data collection and analysis and writing the paper, and manuscript preparation and submission. Rob Melis, Julia Sibiya and Gemechu Keneni involved in supervision of the research work. Bodo Raatz involved in genotyping data analysis and writing the manuscript. Amelework Assefa involved in data analysis and writing the manuscript. Clare Mukankusi involved in designing and performing the experiments and writing the manuscript. Berhanu Fanta involved in designing and performing the experiments and writing the manuscript. Selamawit Ketema assisted on data analysis. Dagmawit Tsegaye assisted on data collection. All authors discussed the results and contributed to the final manuscript.

## Supporting information


**Table S1.** Genotypic information of the 83 resistant genotypes and parental lines
**Figure S1.** Cluster plot which shows the fluorescence values for each genotype

## Data Availability

The data that support the findings of this study are available from the corresponding author upon reasonable request.
