# Supplementary material for: Introgression of bruchid ( Zabrotes subfasciatus) resistance into small red common bean ( Phaseolus vulgaris ) background and validation of the BRU_00261 (snpPV0007) resistance marker
Source: Plant Breed. 2021 Oct 9;140(6):1081–9. doi: 10.1111/pbr.12969 (PMC9293403; doi:10.1111/pbr.12969)
Supplement: Supplementary file 1 — Table S1. Genotypic information of the 83 resistant genotypes and parental lines Figure S1. Cluster plot which shows the fluorescence values for each genotype [file PBR-140-1081-s001.docx]

Supplementary Table 1. Genotypic information of the 83 resistant genotypes and parental lines

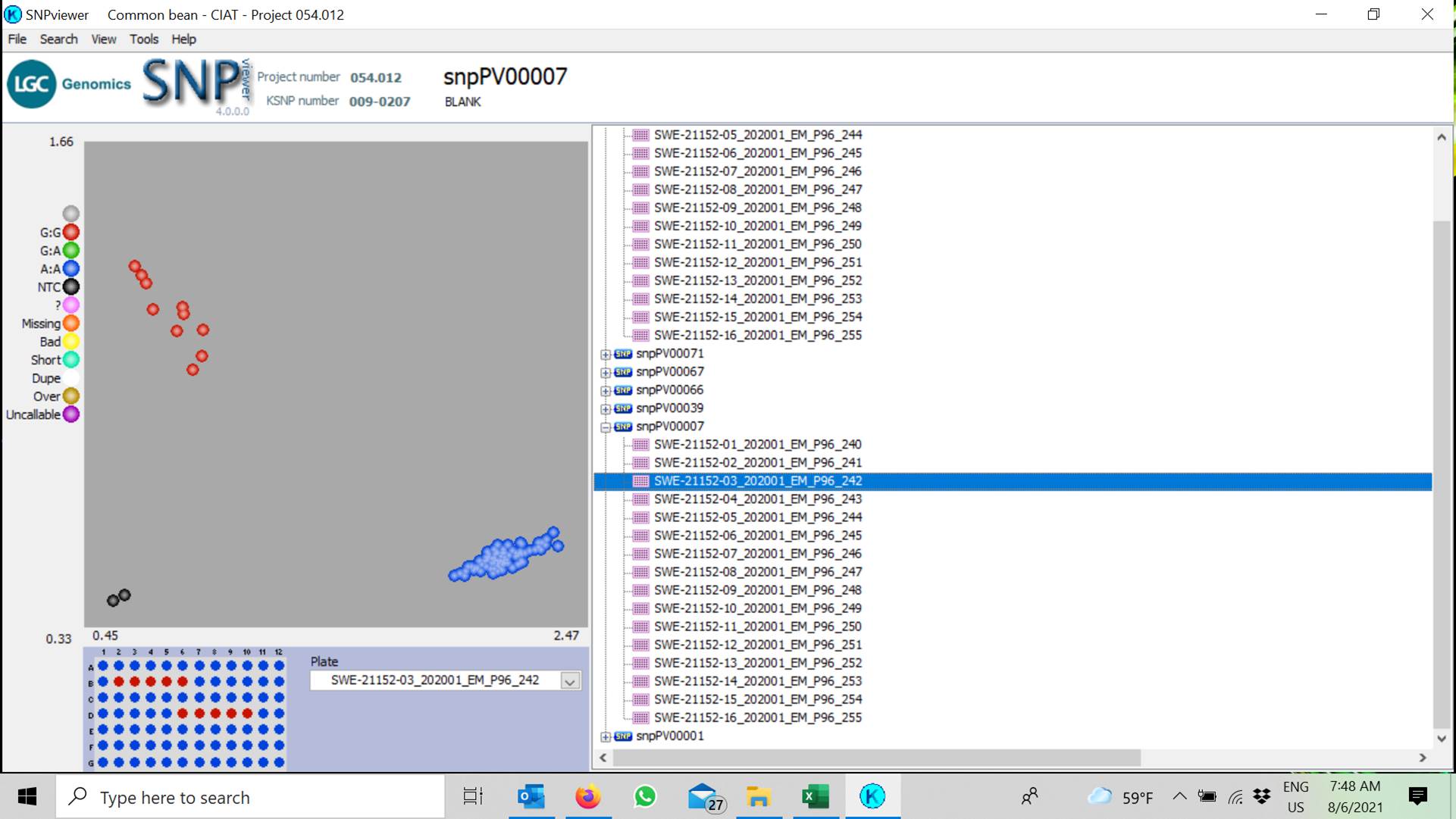


Supplementary Figure 1. Cluster plot which shows the fluorescence values for each genotype
